# Persistence of the Sciatic Artery: A Case Report of a Combined (Complete and Incomplete) Type Causing Leg Ischemia

**DOI:** 10.1155/2012/196798

**Published:** 2012-09-02

**Authors:** Beatrice Cavallo Marincola, Alessandro Napoli, Michele Anzidei, Eugenio Marotta, Fabrizio Boni, Gaia Cartocci, Luca Bertaccini, Vincenzo Noce, Maria Antonietta Pacilé, Carlo Catalano

**Affiliations:** ^1^Department of Radiological, Oncological and Anatomo-Pathological Sciences, Policlinico Umberto I, Sapienza University of Rome, Viale Regina Elena, 324-00161 Rome, Italy; ^2^Department of Surgical Sciences “F. Durante”, Policlinico Umberto I, Sapienza University of Rome, Viale Regina Elena, 324-00161 Rome, Italy

## Abstract

Persistent sciatic artery is a very uncommon embryologic vascular variant, with a prevalence of 0.05% based on angiographic studies. Two different types of this anomaly can occur, complete or incomplete, on the basis of the relationship between sciatic artery and femoral artery. Although many of these patients are asymptomatic, it may represent a threat to the viability of the lower extremity because of atherosclerotic degeneration resulting in aneurysmal dilatation, occlusive thrombosis, or embolic phenomena with distal complication. We present a case of a 64-year-old man with combined, complete and incomplete, type of persistent sciatic artery causing ischemic ulcer of the first toe.

## 1. Introduction

Persistent sciatic artery (PSA) is a rare congenital vascular anomaly, firstly described in 1832 by Green and occurring in about 0.05% of the population [1].

In the majority of cases patients are asymptomatic, but they can develop symptoms if complications (such aneurysm or vessel thrombosis) occur.

## 2. Case Presentation

**Figure 1:**
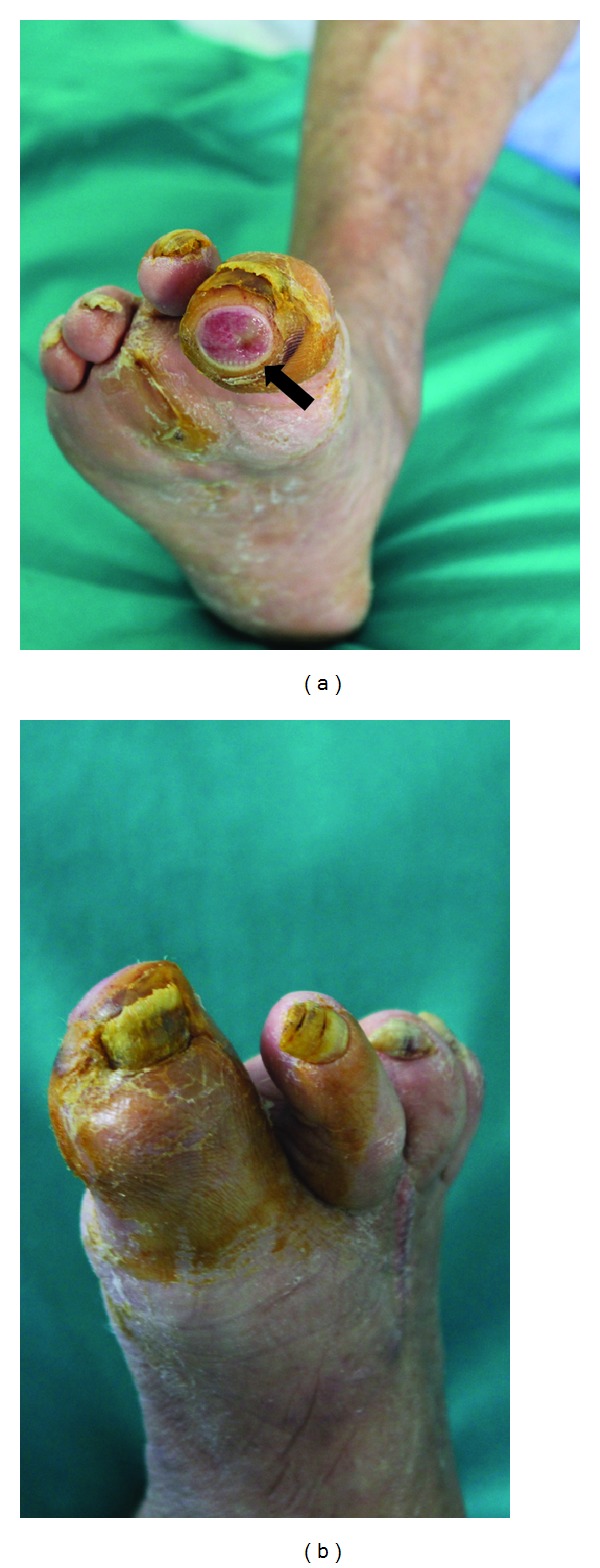
Photographs of dorsal (a) and plantar (b) ischemic toes of the right foot; the I toe presented an ulceration located at the extremity (arrow). Previous amputation of the III toe is also evident.

A 64-year-old man presented to our attention with ischemic signs on the I and II fingers of the right foot and a rounded-shaped ulcer at the top of the I toe (Figure 1). Physical examination found regular femoral pulse but absent tibial pulses. The clinical history evidenced a previous amputation of the III toe but did not explain the primary cause of this injury and there were not any radiological previous examinations. Patient was an exsmoker from 8 years, he suffered of mild hypertension that was pharmacologically controlled; clinical characteristics are summarized in Table 1. The patient reported a long history of diabetes mellitus but under medical therapy since three years that was suspected as the cause of ischemic ulcer; thus, Doppler ultrasound (DUS) examination was required. DUS identified a tiny right superficial femoral artery whose caliber was progressively reducing, in particular at the III medium of the thigh; thus obstruction of this vessel was suspected and Computed Tomography Angiography (CT-A) was required in order to evaluate the vascular axis of the entire lower limb and to plan an optimal treatment.

CT-A was performed from the abdominal aorta to the feet using a Dual Source scanner (Somatom Definition) with the following technical parameters: 200 modulated effective mAs, 100 kVp, 64 × 2 × 0.6 collimation, 1 mm slice thickness, 0.6 recon increment.

A 110 mL bolus of high iodine concentration (400 mgI/mL) was injected at a rate of 4.5 mL/s followed by 40 mL of saline flush injected at the same rate. Postprocessing techniques were used to better evaluate the whole peripheral circulation and included Maximum Intensity Projection, Volume Rendering, and Curved Planar Reformation with and without bone subtraction.

**Figure 2:**
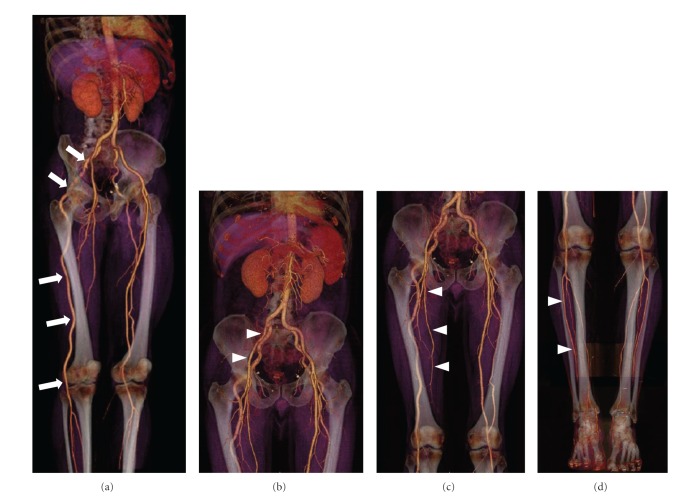
(a) Volume Rendering reconstruction of CT-A study. On the right side a large abnormal artery originates from a hypertrophic internal iliac artery and run along the gluteal region and the posterior thigh supplying the entire vascular axis of the leg (arrows). ((b)-(c)) The external iliac, the deep femoral and the superficial femoral arteries showed a slightly reduced caliber but no obstruction (arrowheads). (d) The circulation of calf and foot are regularly patent (arrowheads).

CT-A images of the right leg demonstrated the presence of a large abnormal artery originating from an hypertrophic internal iliac artery that ran along the gluteal region and the posterior thigh and supplied the entire vascular axis of the leg. The external iliac, the deep femoral, and the superficial femoral arteries showed a slightly reduced caliber but no obstruction; all these vessels ended in thin muscular branches. The distal circulation supplied by the abnormal artery was not compromised: in fact the whole leg vasculature was preserved with normal caliber of anterior tibial, posterior tibial, and peroneal arteries (Figure 2).

**Figure 3:**
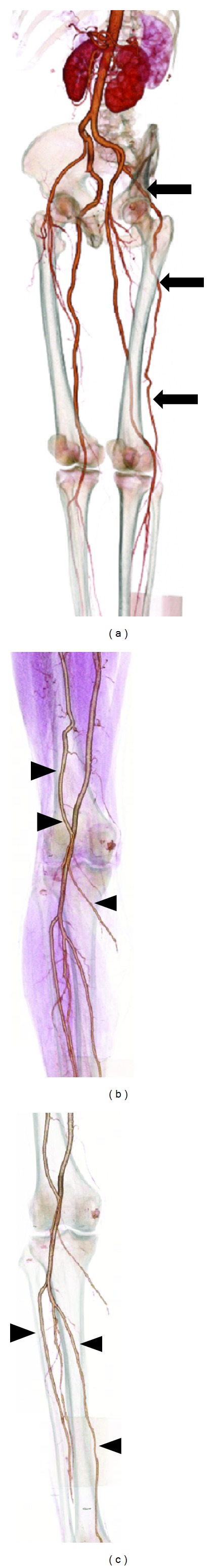
(a) Volume rendering reconstruction of CT-A study. On the left side the same abnormal vessel was depicted (arrows); however, it ended with a progressively reduced caliber at the popliteal level with muscular branches (b) (arrowheads). (c) The distal arterial circulation of the calf and foot was supplied by the superficial femoral artery that presented regular caliber and course (arrowheads).

Similarly, the abnormal artery was depicted also on the left side, presenting the same origin and course (Figure 3). In this leg, however, the artery presented a caliber slightly lower compared to the contralateral one and ended at the level of the knee with muscular branches. The distal arteries of the calf were supplied by the superficial femoral artery that presented regular caliber. The dorsal and plantar arterial circulation was bilaterally regular and patent.

No significant signs of atherosclerosis were depicted in the whole peripheral circulation, even if the patient has diabetes for a long time.

**Figure 4:**
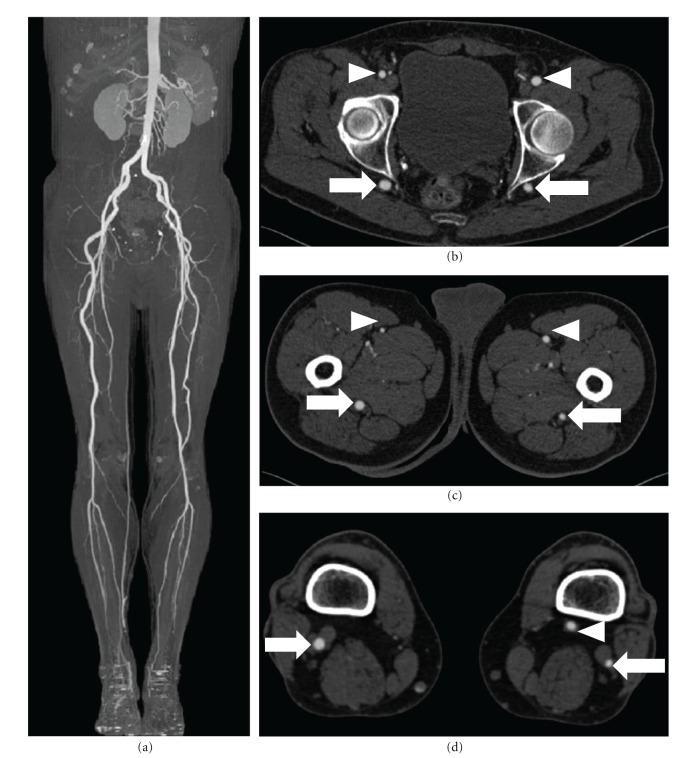
(a) Maximum intensity projection reconstruction on a coronal plane with bone subtraction technique. No severe signs of atherosclerosis were depicted in the whole peripheral circulation. ((b), (c), (d)) Multiplanar reconstructions on axial plane show, respectively, the course of the sciatic artery (arrows) and that of femoral arteries (arrowheads).

CT-A findings were finally compatible with bilateral persistence of sciatic artery with complete type on the right side and incomplete type on the left (Figure 4).

## 3. Discussion

Bilateral occurrence of PSA is rarely encountered, with an incidence of 12%; moreover, even if this type of anomaly have been defined in several reports and vascular pathologies related with PSA have been already encountered and defined, to our knowledge this is the fourth case of combined type (right complete and left incomplete form) of PSA diagnosed from 1964 to 2007 on a total number of 109 living patients [2].

During the first three months of embryonic development the sciatic artery represents the primitive axial artery of the lower limb that normally regresses to form the proximal part of the inferior gluteal artery as long as the femoral artery and the external iliac artery develop. In rare cases the sciatic artery continues existing as a continuation of a hyperplasic iliac artery.

Among many different classifications [3–5], the most commonly used differentiates a complete and an incomplete type on the basis of the relationship between sciatic artery and femoral artery [6]. In the complete type, the most common appearance, the persistent sciatic artery runs to the popliteal artery representing the dominant supply for the lower limb while the superficial femoral artery is hypoplastic and provides only collateral vessels to the lower limb. In the incomplete type, the persistent sciatic artery is hypoplastic and the superficial femoral artery is the main blood supply for the lower limb.

A pathognomonic clinical sign for a PSA is the “Cowie's sign” represented by a diminished or absent femoral pulse in combination with a palpable popliteal pulse [7].

The detection of this anomaly is usually accidental or secondary to complications, such as vascular insufficiency with its signs. Many patients with persistent sciatic arteries remain asymptomatic and do not require treatment. The patient's physical examination can show a pulsatile gluteal mass; symptoms may be pain or may simulate sciatica (when the abnormal artery lies within the sciatic nerve sheath) [8].

The development of aneurysm represents the most common complication, due to early atherosclerotic degeneration and often located at the level of the great trochanter in the gluteal region. Although the etiopathogenesis is not completely known, there may be mechanical (traumatic) and intrinsic causes [9]; hypoplasia of the elastic components may be an important factor of this wall degeneration [10].

Although aneurysmal degeneration appears to be the most frequent (14–38% of incidence) and most serious complication, because of possible vessel rupture, sciatic nerve compression, thrombosis, or embolic phenomena with limb ischemia [11], other possible complications are limb hypertrophy/atrophy, varicosities of the leg, arterio-venous malformations.

Noninvasive procedures such as Doppler-US, CT-angiography, or MR-Angiography represent a useful tool for diagnosis. Radiological investigations are very useful to identify, classify and evaluate treatment options. Doppler US is the first noninvasive modality to confirm the presence of PSA and its complications [12]. CT-angiography is superior to US for the assessment of the whole and bilateral peripheral circulation, for the correct classification of the disease and for the evaluation of possible coexisting complications. MR-angiography is another valid imaging modality that can give similar finding to CTA, with particular attention to the relation between the sciatic artery and the sciatic nerve. Finally, although conventional angiography is considered the gold standard for PSA diagnosis, the selective injection of contrast medium in the external iliac artery may cause an incorrect diagnosis of vessel occlusion due to the lack of enhancement of the popliteal artery [13].

Our patient had only few CTA signs of atherosclerosis, namely, rare, small and calcified plaques considered stable; thus, we couldn't identify any macroscopic cause that could explain the ischemic symptoms; CTA study, however, visualized only the abdominal aorta and peripheral vessel, thus we couldn't exclude the presence of detachment of thrombi from the thoracic aorta. Moreover, from an anatomical point of view, the sciatic artery runs posterior in the gluteal and thigh regions and thus can result stretched from these structures [14], determining reduced blood flow to the extremities. Finally, an ischemic ulcer due to neuropathic origin was also considered as possible cause of leg ischemia, but it was excluded with an electromyography. As matter of fact, the patient had serious risk factors for atherosclerotic disease, thus although the anatomical description may be explained by the persistent sciatic artery, the symptoms may still be the consequence of underlying atherosclerotic disease that is accelerated by risk factors. Particularly, even if we did not depict any macroscopic signs of atherosclerosis, we cannot exclude microvascular disease of the extremities, not detectable with standard diagnostic imaging examinations.

The treatment of PSA is different according to type and symptoms: it may vary from a simple “wait and watch” approach to bypass or other surgical revascularization techniques to major/minor amputation. In fact, an asymptomatic persistent sciatic artery does not require specific management, but it has to be monitored to prevent the risk of thromboembolic complications thus avoiding limb amputation. Contrarily, in symptomatic cases an endovascular/surgical treatment may be appropriate, especially if aneurysm or arterial thrombosis is present.

In our case, vascular surgeons were required to perform minor amputation of the first toe; a surgical or endovascular revascularization was not chosen because of the absence of vessel aneurysm, stenosis, or thrombosis; finally the clinicians in consensus with the vascular surgeons decided to perform a conservative local treatment of the ischemic signs of the second toe.

## Figures and Tables

**Table 1:** Patient's clinical characteristics.

Age	69 years old
Gender	Male
Body mass index	23
Smoke	Ex (from 8 years)
Diabetes	Type 2, recently controlled with pharmacological therapy
Hypertension	Present, well controlled with pharmacological therapy
Dyslipidemia	Absent
